# Towards Understanding the Roles of Heparan Sulfate Proteoglycans in Alzheimer's Disease

**DOI:** 10.1155/2014/516028

**Published:** 2014-07-23

**Authors:** Gan-lin Zhang, Xiao Zhang, Xiao-min Wang, Jin-Ping Li

**Affiliations:** ^1^Beijing Hospital of Traditional Chinese Medicine, Capital Medical University, Beijing 100010, China; ^2^Department of Neuroscience, Pharmacology, University of Uppsala, The Biomedical Center, 751 23 Uppsala, Sweden; ^3^Department of Medical Biochemistry and Microbiology, University of Uppsala, The Biomedical Center, 751 23 Uppsala, Sweden

## Abstract

Alzheimer's disease (AD) is the most common form of dementia, characterized by progressive loss of memory and cognitive dysfunctions. A central pathological event of AD is accumulation and deposition of cytotoxic amyloid-*β* peptide (A*β*) in the brain parenchyma. Heparan sulfate proteoglycans (HSPGs) and the side chains heparan sulfate (HS) are found associated with A*β* deposits in the brains of AD patients and transgenic animal models of AD. A growing body of evidence from *in vitro* and *in vivo* studies suggests functional roles of HSPG/HS in A*β* pathogenesis. Although the question of “how and why HSPG/HS is codeposited with A*β*?” still remains, it is within reach to understand the mechanisms of the events. Recent progress by immunohistochemical examination with advanced antibodies shed light on molecular structures of HS codeposited with A*β*. Several recent reports have provided important new insights into the roles of HSPG in A*β* pathogenesis. Particularly, experiments on mouse models revealed indispensible functions of HSPG in modulating A*β*-associated neuroinflammation and clearance of A*β* from the brain. Application of molecules to interfere with the interaction between HS and A*β* peptides has demonstrated beneficial effects on AD mouse models. Elucidating the functions of HSPG/HS in A*β* deposition and toxicity is leading to further understanding of the complex pathology of AD. The progress is encouraging development of new treatments for AD by targeting HS-A*β* interactions.

## 1. Introduction

*Structure of Heparan Sulfate Proteoglycans*. Heparan sulfate proteoglycans (HSPGs) are heavily glycosylated proteins, in which several heparan sulfate (HS) glycosaminoglycan (GAG) chains are covalently attached to a core protein. HSPGs are expressed on the cell surface and in the extracellular matrix (ECM) in all tissues. Cell surface HSPGs are membrane-spanning syndecans (SDCs) and lipid-anchored glypicans (GPCs). There are four members in SDC family (SDC 1–4) and six in GPC family (GPC 1–6). Secreted HSPGs are agrin, collagen type XVIII, and perlecan [1]. HS polysaccharide chains are characterized by highly structural heterogeneity with respect to the chain length and sulfation pattern, generated by a complex biosynthetic process within the Golgi apparatus [2, 3]. Functions of HSPGs are mainly attributed to the HS side chains that interact with a spectrum of protein ligands including growth factors, cytokines, enzymes, lipase, apolipoproteins, and protein components of the ECM, exerting biological activities in development, homeostasis, and diseases [3, 4].

The diverse functions of HS in different biological settings have been extensively studied, and substantial information is obtained. One of the most studied molecular mechanisms of HS is in signal transduction process, particularly growth factor medicated signaling. For example, HS mediates high affinity binding of fibroblast growth factor-2 (FGF-2) to its receptor promoting the formation of a stable tertiary signal complex of FGF-2-HS-FGF-2 receptor [5]. Apart from mediating growth factor activities, HS also functions as coreceptors in other biological activities, for example, modulating the interaction of neuropeptide agouti-related protein with melanocortin receptors 3 and 4 (MC3R and MC4R) in the hypothalamus and regulating food consumption [6–8]. Moreover, membrane HSPGs also act as endocytic receptors for diverse macromolecules such as lipid, growth factors, receptor ligands, and morphogens [1, 9].

Secreted HSPGs, agrin [10] and perlecan [11], constitute major structural molecules in the ECM and basement membrane (BM) along with collagens and other proteins (for review, see [12]). In the ECM, HSPG serves as storage for a number of molecules, such as growth factors and chemokines. In addition, HSPG also plays important roles in maintaining the integrity of ECM and BM [13, 14] and modulating cell mobility [15–17] (also for review, see [4]). In the BM, HSPG, along with collagen IV and laminin-entactin/nidogen complex, controls blood vessel permeability and takes a part in transportation of solutes between vessels and ECM [18, 19]. The ultrastructure of BM can be changed in disease conditions [20] and aging [21], probably due to abnormal production and breakdown of BM components including HSPGs [20].

*Heparanase*. Heparanase is an endo-*β*-glucuronidase that specifically cleaves HS side chains of HSPG, releasing oligosaccharide products at the size of 4–7 kDa (10–20 sugar units) [22]. Heparanase is normally expressed at a low level in majority of tissues including the brain [23]. Surprisingly, this unique HS-specific glycosidase is not essential for animal development and homeostasis, as demonstrated by targeted interruption of the heparanase gene in mouse [24]. The heparanase null mice produce longer HS chains in comparison to wildtype mice; however, there is no accumulation of the polysaccharide in organs, indicating that heparanase is not an indispensible enzyme for HS catabolism. In contrast, overexpression of heparanase in mice resulted in extensive modification of HS chains, producing short fragments with increased sulfation that exert higher potency for FGF-2-HS-FGF-2 receptor resembling [25]. This makes the heparanase transgenic mouse (Hpa-tg) a valuable tool for study of HS functions in different diseases [26–29]. Changes in expression of heparanase in tissues, mainly upregulation, have been reported in several diseases, particularly in cancers [30]. Increased expression of heparanase is detected in brain tumor glioma tissues from human and animal models, where heparanase is suggested to play an important role in the control of tumor cell proliferation and invasion [31]. Cerebral ischemia markedly increased heparanase levels in endothelial cells and astrocytes of mouse [32] and rat [33] brains. Available information suggests that heparanase may function as a regulatory factor in different pathological conditions, including tumor and inflammation, exerting its functions through modification of HS structure [34]. Moreover, heparanase has been shown to have nonenzymatic activities, most likely through direct interaction with cell surface receptors, which needs further investigations [35].

*Aβ* *Pathology of Alzheimer's Disease*. Alzheimer's disease (AD) is a major central nervous system disease characterized by a progressive neurodegeneration with a clinical phenotype of cognitive impairment. A histopathological hallmark of AD is extracellular A*β* deposition in brain parenchyma manifested as senile A*β* plaques [36]. The pathological A*β* peptides of 40 or 42 amino acids are products of sequential cleavage of the amyloid *β* precursor protein (A*β*PP), a transmembrane glycoprotein, by *β*-secretase (*β*-site APP cleaving enzyme 1: BACE1) [37] and *γ*-secretase, a multisubunit protease complex composed of at least 4 proteins including presenilin 1 and 2 [38]. Deposition of A*β* in the brain is attributed to excessive accumulation and aggregation of A*β* in the brain. Accumulation and deposition of A*β* most probably resulted from overproduction in the brain or/and impaired removal of A*β* from the brain [39]. Autosomal dominant mutations in three genes, that is, A*β*PP gene (*APP)* and presenilin 1 and 2 genes (*PSEN1* and* PSEN2*), can cause early onset familial AD, accounting for <10% of AD cases [40–42]. All these mutations can result in overproduction of the A*β* peptides, leading to their accumulation and aggregation in the brain [43–45]. In clinic, the most common form of AD is late-onset sporadic AD accounting for about 90% of AD cases. Sporadic AD is not associated with genetic mutations, and no overproduction of A*β* was found. In these cases, it is generally believed that overall A*β* clearance is impaired, resulting in accumulation of A*β* peptides [46, 47]. In the brains of AD patients and some aging individuals with no clear diagnosis of dementia, A*β* is found to accumulate and deposit in blood vessel walls, named cerebral amyloid angiopathy (CAA), which has been interpreted as a sign of impaired A*β* clearance from the brain [48].

There are several ways for A*β* clearance, including degradation by proteolytic enzymes [49], receptor mediated A*β* transport across the blood-brain barrier (BBB) in which the main receptor is low-density lipoprotein receptor related protein-1 (LRP-1) [50], phagocytosis by innate immune cells (macrophages) [51], and perivascular drainage along the BM of blood vessels [52].

## 2. Interaction of HS with A***β***

Several* in vitro* studies demonstrate interaction of A*β* with GAGs including HS and heparin (a HS analogue with higher sulfation degree) [53–56]. It has been found that the HHQK domain at the N-terminus of A*β* is a HS binding motif and this sequence has also been shown to bind microglial cells, suggesting that microglia interact with A*β* through membrane associated HS [57]. Concurrently, a HS sequence of *N-*sulfated hexasaccharide domain containing critical 2-O-sulfated iduronic acid residues binds fibrillar A*β* and was identified in human cerebral cortex. Interestingly, this HS domain also serves as a binding site for the neuroprotective growth factor FGF-2. This evidence suggests that, in AD brain, neurotoxic A*β* may compete with neuroprotective FGF-2 for a common HS binding site [58]. Affinity of HS binding to A*β* is associated with its sulfation pattern, as heparin shows a higher affinity to A*β*, while desulfated HS essentially lost binding capacity to A*β*. This interaction is also dependent on chain length of the GAGs, as heparin fragments shorter than 6-sugar units do not bind to A*β* [58]. Furthermore, it has been proposed that the A*β*-HS interaction is mutually protective, such that HS is protected from heparanase degradation [53] and A*β* is protected from protease degradation [59].

## 3. Codeposition of HS with A***β*** in AD Brain—Updated Findings

The presence of glycosaminoglycans (GAGs) in A*β* plaques in AD brain was first identified using Congo red staining for A*β* fibrils and Alcian blue dye for sulfated GAGs in brain sections of autopsy specimens of AD patients about 30 years ago [60]. The presence of HSPGs in A*β* plaques and CAA was later revealed by immunostaining with specific antibodies recognizing the core proteins of HSPGs [61–63]. With these antibodies, subtypes of HSPGs including SDC 1–3, GPC 1, and agrin have been immunolocalized in A*β* plaques and CAA of AD brains [64, 65]. Development of antibodies recognizing different A*β* fragments further promoted characterization of interaction between A*β* and HS.

Recent studies employed advanced type of anti-HS antibodies that differentially recognizes certain structures of HS polysaccharide chains [66, 67]. For example, phage display antibodies EV3C3 and HS4C3 recognize fully N-sulfated motifs in HS chain, while RB4EA12 and HS4E4 recognize partially N-sulfated and N-acetylated HS motifs [66, 68, 69]. Availability of these unique antibodies allowed us to analyze the molecular structure of HS codeposited with A*β* in the brain. By costaining the AD brain sections with an anti-HS phage display antibody HS4E4 and antibodies specific for A*β* species, we found that HS is differentially deposited with A*β*40 or A*β*42 in neuritic and diffuse plaques [70]. In sporadic AD cases, HS4E4 immunosignals are preferentially colocalized with A*β*40 in the cores of senile plaques; however, the HS4E4 signals are absent from A*β*42-rich diffuse deposits. In a recent study, antibodies (EV3C3 and HS4C3) recognizing highly N-sulfated HS detected strongest immunosignals in both fibrillar and nonfibrillar A*β* plaques, while antibodies (RB4EA12 and HS4E4) recognizing HS regions with lower degree of N-sulfation only stained fibrillar A*β* plaques [68], indicating a distinct property of HS structures in interaction with different A*β* aggregates* in vivo*. These reports are in agreement with our findings, confirming that only fibrillar A*β* plaques of A*β*40 deposits are colocalized with lower sulfated HS motifs. We have identified the membrane bound HSPGs, GPC 1, and SDC 3 in glial cells associated with A*β* deposits in dense core plaques, proximal to sites of HS accumulation, and suggested that HS codeposited with A*β*40 in neuritic plaques is mainly derived from glial cells [70]. RB4CD12 is another phage display antibody that recognizes highly sulfated domains of HS [71]. This antibody strongly stained both diffuse and neuritic A*β* plaques in the brains of AD and several transgenic AD mouse models. Interestingly, the RB4CD12 epitope accumulated in A*β* plaques can be demolished by extracellular sulfatases (Sulf-1 and Sulf-2)* ex vivo* [72], suggesting that 6-O-sulfated glucosamine residues are within the HS sequence interacting with A*β*.

These recent findings of selective deposition of HS with different species and forms of A*β* strongly suggest distinct roles of HS in A*β* aggregation and deposition. These studies point that HS/HSPG constitutes a part of A*β* plaques and the findings support the notion that HS plays a role in A*β* plaque formation and persistence.

## 4. HS Mediated A***β*** Uptake—Implications in A***β*** Cytotoxicity and Clearance

In the brain, A*β* are present in both extracellular and intracellular pools and extracellular A*β* contributes to intracellular A*β* through internalization mechanisms [73]. Cell types in the brain are known to engulf A*β* including neurons, endothelial cells [74], smooth muscle cells [75], and glial cells (microglia and astrocytes) [76, 77]. Internalization of A*β* into cells has been shown to be associated with A*β* cytotoxicity [78, 79]. Several cell surface macromolecules of microglia/macrophages are reported to play roles in A*β* uptake, including toll-like receptor [80], complement receptors [81], scavenger receptors [76, 82], LRP-1 [83], and transmembrane protein CD33, a member of the sialic acid-binding immunoglobulin-like lectins [84] (also for review, see [85]). HSPG functions as a cell surface receptor for entry of diverse macromolecules into cells; in this context, both the core protein and the HS side chains of HSPG are attributed to regulation of endocytosis (for review, see [9]). Having this in mind, we studied A*β*40 uptake and associated toxicity in Chinese hamster ovary (CHO) cell lines. After exposure to A*β*40, the CHO wildtype cells (CHO-WT) survived poorly, whereas the HS-deficient CHO pgsD-677 cells were resistant to the treatment. In correlation with A*β* cytotoxicity, the added A*β*40 was substantially uptaken by CHO-WT but barely by CHO pgsD-677 cells [86]. Likewise, A*β*40 cytotoxicity was attenuated in human embryonic kidney cells (HEK293) overexpressing heparanase due to extensive degradation of HS chains [86]. These findings suggest that cell surface HS mediates A*β* internalization and toxicity.

According to “amyloid hypothesis,” the cause of the majority form of AD, that is, late-onset sporadic, is due to impaired clearance of A*β* from the brain [47, 87]. Transport of A*β* across the BBB from brain to blood is an important route for A*β* clearance, where transcytosis requires A*β* to attach to cell surface after which it is internalized and subsequently released at the luminal side of the endothelium. LRP-1 at the surface of blood vessel endothelial and smooth muscle cells has been reported to function as A*β* cargo in this process [50, 75]. It has been recently reported that LRP-1 and HSPGs mediate A*β* internalization in a seemingly cooperative manner, in which HSPG is more important for A*β* binding to cell surface than LRP-1 [88]. Another important player in this context is apolipoprotein E (ApoE). ApoE and HS are consistently codetected in A*β* deposits and have been ascribed various roles in the pathogenesis of AD [89, 90]. ApoE can bind to HSPG forming functional complex of ApoE/HSPG; alternatively, it joins HSPG/LRP-1 uptake pathway in which ApoE first binds to HSPG and then presents to LRP-1 for uptake (for review, see [91]). The finding of codistribution of ApoE, HS, and LRP1 in A*β*40-positive microvasculature in the hippocampus of individuals with Down's syndrome (DS), diagnosed with AD, encouraged us to investigate correlation of these molecules in A*β* uptake and clearance [92]. We investigated the functional relationship between A*β* and ApoE and their interactions with cell surface HS and LRP-1 [92]. Coincubation of A*β* with CHO cells either deficient in HS (CHO pgsD677) or in LRP-1 (CHO 13-5-1) along with CHO-WT revealed that addition of ApoE in the cell culture increased A*β* association to the cells, which is dependent on presence of HSPG and LRP-1 on the cell surface. ApoE uptake by the cells does not require presence of both HSPG and LRP1; however, lack of HS in the CHO pgsD677 cells resulted in aberrant intracellular ApoE processing. These data propose that the complex interactions of ApoE, LRP-1, and HSPG facilitate A*β* internalization, which may represent one of major routes for A*β* clearance through transportation of ECM A*β* across BBB into the vessel lumen [92].

## 5. Heparanase in Aging and AD—Implications in Transmigration of Blood-Borne Monocytes

**Figure 1:**
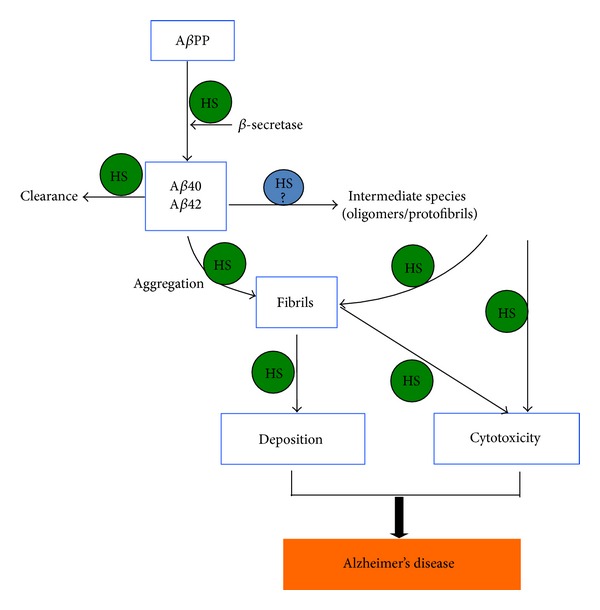
Heparan sulfate (HS) is involved in essentially each step of amyloid-*β* (A*β*) pathological development in Alzheimer's disease. HS modulates *β*-secretase (BACE) activity and accelerates A*β* aggregation and fibrillization. It is unclear whether HS is involved in formation of the toxic oligomers/protofibrils; however, HS mediates toxic effect of different types of A*β* fibrils. HS in the basement membrane participates in clearance of A*β*.

Heparanase expression in the brain is at marginally detectable level [23, 29], while, in several pathological conditions of the brain, expression of heparanase has been found elevated [31, 32]. Although limited information is available regarding the impact of heparanase on AD pathogenesis, A*β*40 has been shown to protect heparanase-catalyzed degradation of HSPGs* in vitro* with predicted effect contributing to the stability and persistence of A*β* plaques [53]. Our recent study has revealed increased vasculature expression of heparanase in the brains of AD patients and a mouse model that overexpresses human A*β*PP (Tg2576 mice) [29]. Since HS is involved in almost every step of A*β* pathogenesis found in AD (Figure 1), it is of great importance to study expression and activity of heparanase in the brain of aging subjects, both human and animal models.

In the brain, perivascular macrophages derived from blood-borne mononuclear cells play an important role in A*β* clearance [51, 93, 94]; A*β* peptides are uptaken and subsequently degraded by proteases [95]. Several* in vivo *studies have demonstrated the multiple functions of HS and heparanase in inflammatory reactions with regard to infiltration of blood-borne immune cells into infected tissues [28, 96]. In this scenario, molecular structures of HS, for example, sulfation pattern and chain length, are pivotal in interaction between endothelial cells and leukocytes as well as with the soluble inflammatory cytokines. Accordingly, we have recently studied the potential roles of heparanase and HS in mediating blood-borne monocytes across blood vessel wall into the brain parenchyma on the transgenic mouse model overexpressing heparanase (Hpa-tg). Overexpression of heparanase resulted in shorter HS chains in the brain of Hpa-tg mouse [29]. In the study, we applied two experimental regimens, that is, localized cerebral microinjection of aggregated A*β*42 and systemic challenge by intraperitoneal injection of lipopolysaccharide (LPS), a bacterial endotoxin. Microinjection of aggregated A*β*42 into the brain elicited an inflammatory response restricted to the injection site of the wildtype mice, characterized by massive infiltration of microglia/macrophages. This inflammatory reaction clearly showed a beneficial effect for clearance of the injected A*β*. In comparison, recruitment and activation of immune cells (microglia and blood-borne monocytes) were significantly attenuated around the injection site of Hpa-tg mouse brain, which resulted in detainment of the injected A*β*42 [29]. The LPS-treated wildtype mice also showed massive activation of resident microglia as well as recruitment of monocyte-derived macrophages in the brain parenchyma, whereas Hpa-tg mice exhibited restricted inflammation with significantly fewer infiltrated macrophages. The mechanism for the reduced recruitment of inflammatory cells into the brain of Hpa-tg mice was verified with an* in vitro* BBB model constituted with primary endothelia cells and pericytes [29].

The integrity of ECM and the capillary vascular basement membrane (VBM) scaffold is often found severely damaged in association with A*β* deposition [97, 98], which may be responsible for perturbed elimination of solutes and A*β* from parenchyma, consequently leading to development of CAA [99]. As HSPGs are major components of the ECM and VBM and heparanase activity is strongly implicated in structural remodeling of the ECM and BM through degradation of HS, heparanase expression may markedly contribute to pathological changes in the ECM and VBM in AD brain, accordingly affecting A*β* clearance. There is essentially no information with this regard and studies are needed to explore the implications of HS in A*β* transportation and clearance.

## 6. Conclusion and Perspectives

Principle treatments for AD with regard to A*β* pathology are to reduce production, improve clearance, and prevent aggregation of the pathological peptides. Considering that HS-A*β* interaction contributes to every stage of the A*β* pathogenesis in AD, including production, clearance and accumulation, aggregation, and toxicity of A*β* (Figure 1), it is rational to hypothesize that interfering HS-A*β* interaction may have multiple beneficial effects. Earlier studies show that treatment with low molecular weight heparin (LMWH) reduced A*β* burden in the brain of an AD mouse model overexpressing human A*β*PP [100]; the effect is probably that the LMWH competes with endogenous HS, blocking the HS-A*β* interaction. This assumption is supported by our findings that the fragmentation of HS by overexpressed heparanase in mouse attenuated deposition of serum A amyloid (SAA; another amyloid protein) [27]. Though it is improper to use LMWH for treatment of AD, it is possible to apply non-anticoagulant LMWH or HS mimetics for the purpose. With the progress in characterization of HS molecular structures dissected from A*β* plaques, it should be possible to design compounds mimic to the HS structures that interact with A*β* to block its aggregation as well as to neutralize its toxicity. Moreover, targeting A*β* producing enzymes, that is, BACE1 and *γ*-secretase, constitutes one of the potential treatments for AD. Interestingly, HSPG has been found to modulate BACE activity [101, 102], and efforts are being made to synthesize HS-oligosaccharides as inhibitors of BACE [103]. In light of experimental and clinical evidences addressing the role of HS in A*β* pathology, it is plausible to expect that novel treatments by targeting HS-A*β* interaction may contribute to AD treatment and to improve effects of other treatments. Apart from designed synthesis of HS mimetics, natural anionic oligosaccharides, such as glycosaminoglycans isolated from marine animals and natural herbs, should also be explored for the potential to be developed as drug candidates for this particular application.
